# Corylifol A from *Psoralea corylifolia* L. Enhances Myogenesis and Alleviates Muscle Atrophy

**DOI:** 10.3390/ijms21051571

**Published:** 2020-02-25

**Authors:** Yeongeun Han, Hyejin Lee, Hua Li, Jae-Ha Ryu

**Affiliations:** Research Institute of Pharmaceutical Sciences and College of Pharmacy, Sookmyung Women’s University, 100 Chungparo 47-Gil, Yongsan-Gu, Seoul 04310, Korea; thddl1428@naver.com (Y.H.); u9698115@naver.com (H.L.); cooldog227@hotmail.com (H.L.)

**Keywords:** *Psoralea corylifolia*, corylifol A, myogenesis, muscle atrophy, C2C12 myoblast

## Abstract

Inflammatory conditions caused by cancer, chronic diseases or aging can lead to skeletal muscle atrophy. We identified myogenic compounds from *Psoralea corylifolia* (PC), a medicinal plant that has been used for the treatment of inflammatory and skin diseases. C2C12 mouse skeletal myoblasts were differentiated in the presence of eight compounds isolated from PC to evaluate their myogenic potential. Among them, corylifol A showed the strongest transactivation of MyoD and increased expression of myogenic markers, such as MyoD, myogenin and myosin heavy chain (MHC). Corylifol A increased the number of multinucleated and MHC-expressing myotubes. We also found that the p38 MAPK signaling pathway is essential for the myogenic action of corylifol A. Atrophic condition was induced by treatment with dexamethasone. Corylifol A protected against dexamethasone-induced myotube loss by increasing the proportion of multinucleated MHC-expressing myotubes compared with dexamethasone-damaged myotubes. Corylifol A reduced the expression of muscle-specific ubiquitin-E3 ligases (MAFbx and MuRF1) and myostatin, while activating Akt. These dual effects of corylifol A, inhibition of catabolic and activation of anabolic pathways, protect myotubes against dexamethasone damage. In summary, corylifol A isolated from *P. corylifolia* alleviates muscle atrophic condition through activating myoblast differentiation and suppressing muscle degradation in atrophic conditions.

## 1. Introduction

A number of internal and external stimuli, including physical injury or inflammatory environments within the body, can induce skeletal muscle atrophy. An increase in the aging population is known to be a primary cause for the increased incidence of skeletal muscle atrophy (referred to as sarcopenia). Aging increases the risk of chronic diseases, including diabetes, obesity, abnormal endocrine regulation, depletion of hormones and cancer. These chronic diseases also cause muscle atrophy (referred to as cachexia). Thus, muscle atrophy is one of the most important diseases that need to be addressed in our increasingly aged society. 

In cachexia and sarcopenia, reductions in the size and strength of muscles makes it difficult to control body movement and results in low quality of life as well as higher mortality [1,2,3,4]. To protect against atrophy, muscles regenerate through the processes of satellite cell activation, myogenesis and muscle protein synthesis. Quiescent satellite cells located beneath the basal lamina can be activated by physical injury or growth stimuli to regenerate muscles [5]. Activated muscle satellite cells proliferate, and myoblasts then express various myogenic factors to initiate myoblast differentiation. The fully differentiated myoblasts, i.e., myotubes, then fuse together to form muscle bundles. Therefore, activators of normally quiescent satellite cells have attracted attention as targets for developing muscle-strengthening drugs or supplements. 

In addition to muscle protein synthesis, muscle degradation is also part of the homeostatic mechanism for maintaining muscle mass and fiber size. In muscle atrophic conditions, protein degradation pathways are upregulated through proteolytic machinery such as ubiquitin-proteasome, caspase, calpain, cathepsin L or autophagy. Potential therapeutic agents and several molecular mechanisms for regulating skeletal muscle atrophy have been recently reviewed [6]. At present, megestrol acetate is the only remedy approved by U.S. FDA for the treatment of AIDS- or cancer-induced cachexia. However, its side effects have increased the need for new drug developments [6]. In 2016, the CDC (Centers for Disease Control and Prevention) newly established an ICD-10-CM code for sarcopenia as an independent medical condition accompanying the age-associated losses of muscle mass, strength and function. At present, there are no FDA-approved drugs for sarcopenia. Considering the reliability and stability of medicinal plants that have long been in use, their application as therapeutic agents for muscle atrophy has been suggested [7]. Indeed, we have reported on several myogenic compounds from medicinal plants that can be used to develop anti-myopathy drugs [8,9,10,11]. 

The seeds of *Psoralea corylifolia* L. (Fabaceae, PC) have been traditionally used for the treatment of several symptoms, including stomachache, diuretic, leprosy, skin diseases such as eczema, leukoderma and psoriasis, in Asian countries [12]. They contain coumarins, flavones, chalcones and meroterpenes, which have antitumor, antibacterial, anti-inflammatory, antimelanogenic and antiosteoporotic activities [13,14,15,16,17,18,19,20]. We previously reported several physiological activities of compounds purified from PC [16,17,19,21,22]. We also found that the major compound of PC, bakuchiol, has potential myogenic activity [23]. In this study, we attempted to identify additional potent myogenic agents that could be derived from PC. Further, we wished to uncover mechanisms by which the myogenic capacity of myoblasts and protective activity of muscle fibers against glucocorticoid-induced damage can be enhanced.

## 2. Results

### 2.1. Extracts of Psoralea Corylifolia Enhance Myogenesis

The myogenic activity of ethanol extracts of *Psoralea corylifolia* L. (EPC) was evaluated in C2C12 myoblasts cultures. Mouse C2C12 cells are derived from muscle satellite cells and can be differentiated into myotubes by cell–cell contact under low serum conditions [10]. Treatment with differentiation medium (DM) triggers differentiation of C2C12 cells, which then gradually convert into long, tubular myocytes to build muscle bundles.

**Figure 1:**
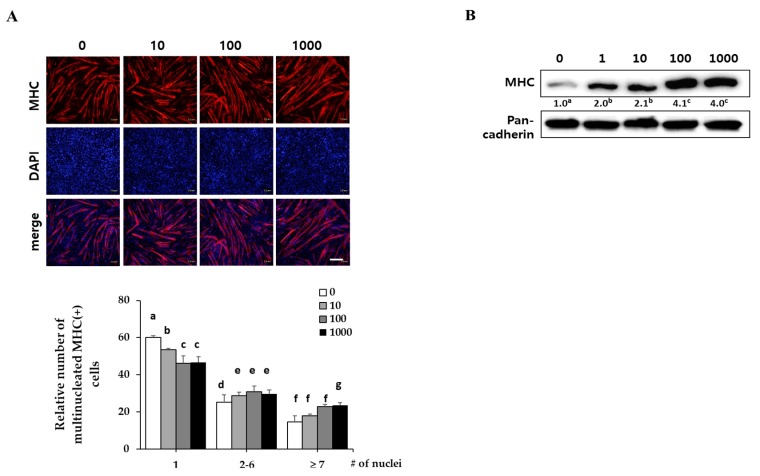
Effect of ethanol extracts of *Psoralea corylifolia* (EPC) on myogenesis. C2C12 cells were supplemented with differentiation medium (DM), including the indicated concentrations of EPC (1, 10, 100 and 1000 ng/mL) for 3 days, and then cells were collected to perform (**A**) immunostaining of MHC (red) and DAPI (blue) (scale bar = 200 µm) and (**B**) Western blot analysis to determine the level of myosin heavy chain (MHC) expression. The level of MHC protein was quantified and normalized to pan-cadherin. Values are mean (*n* = 3). The images are representative of three independent experiments with similar results. Means without a common superscript differ significantly (*p* < 0.05).

C2C12 myoblasts were differentiated in DM with the indicated concentrations of EPC (1, 10, 100 and 1000 ng/mL) for three days. They expressed myosin heavy chain (MHC) as a terminal myogenesis marker [10] that can be observed with immunostaining for MHC (red) and 4′-6-diamidino-2-prenylindole (DAPI) (blue). Immunostaining results showed that EPC increased the numbers of cylinder-shaped and multinucleated myotubes in a dose-dependent manner (Figure 1A). Western blot analysis revealed that EPC dose-dependently increased MHC expression of myotubes up to 3.5-fold, as compared with the control (Figure 1B). Pan-cadherin was used as a loading control. 

**Figure 2:**
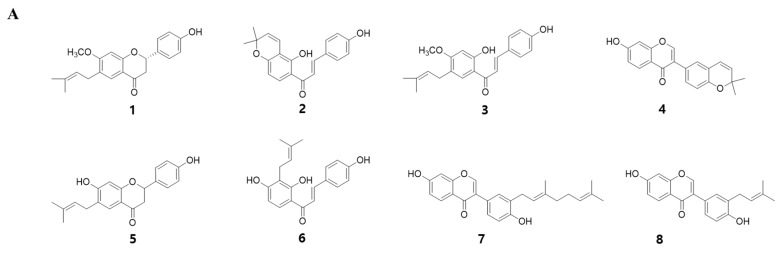

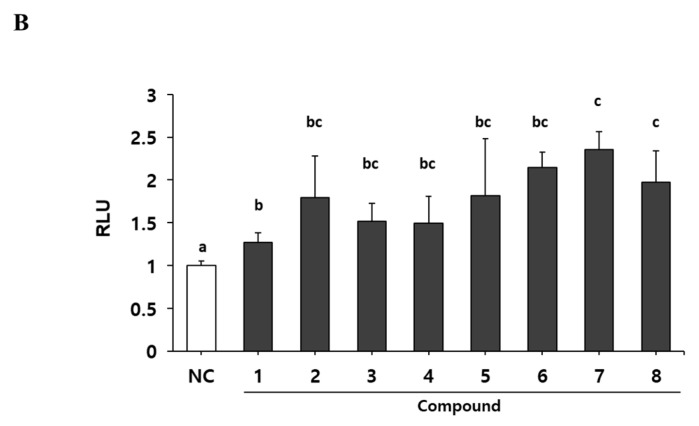
Myogenic effect of compounds **1**–**8** isolated from PC. (**A**) Structures of **1**–**8**. (**B**) Transiently transfected C2C12 cells with a MyoD-responsive reporter gene were differentiated in the presence of each compound (100 nM), followed by measurement of luciferase activity. Data are expressed as mean ± S.D. of three independent experiments. RLU: relative luciferase units. NC: negative control (DMSO) and **1**–**8**: compounds **1**–**8**. Means without a common superscript differ significantly (*p* < 0.05).

To identify the myogenic compounds from EPC, we purified five flavonoids and three chalcones and identified their structures as being bavachinin (**1**), isobavachromene (**2**), bavachalcone (**3**), corylin (**4**), bavachin (**5**), isobavachalcone (**6**), corylifol A (**7**) and neobavaisoflavone (**8**) by spectroscopic data analysis (Figure 2A) [24,25,26,27,28]. We measured endogenous transcriptional activity of MyoD in C2C12 cells to evaluate the myogenic potential of the purified compounds. MyoD is required for terminal specification in muscle cell lineages and functions as a transcriptional factor to induce the expression of myogenic regulatory factors [29]. All of the compounds purified from PC significantly increased MyoD transcriptional activity in myoblasts. Among them, corylifol A (**7**) was highest and increased MyoD transactivation by 2.3-fold, as compared with the control (Figure 2B). Therefore, we chose corylifol A as the most potent myogenic component from PC and carried out the following experiment to clarify its myogenic potential and underlying mechanisms.

### 2.2. Corylifol A Promotes Myogenesis

**Figure 3:**
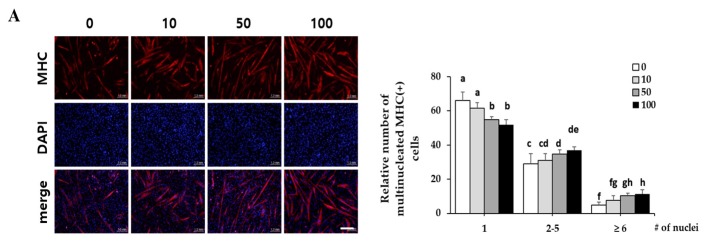

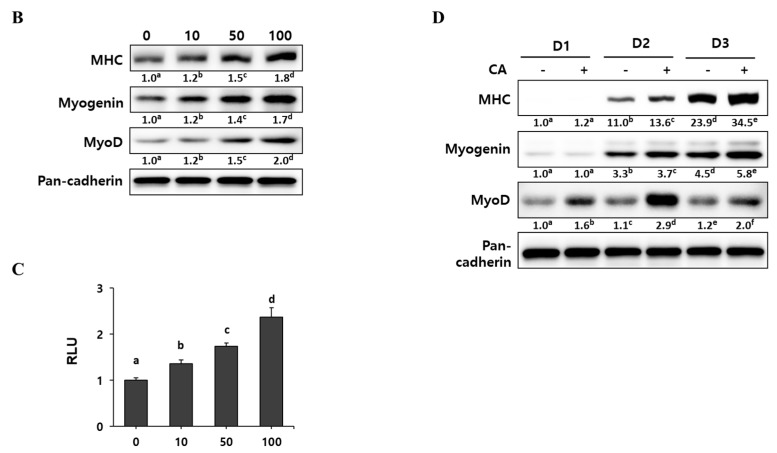
Effect of corylifol A on myogenesis. C2C12 cells were differentiated in the presence of corylifol A (CA, 10, 50 and 100 nM) for 3 days, and then cells were collected to perform (**A**) immunostaining of MHC (red) and DAPI (blue) (scale bar = 200 µm) and (**B**) Western blot analysis to determine the expression of myogenic markers. Values are mean (*n* = 3). (**C**) C2C12 myoblasts were transfected with a MyoD-responsive reporter 4RTK-luciferase, and then cells were differentiated in the presence of corylifol A, followed by measurement of luciferase activity. Data are expressed as mean ± S.D. of three independent experiments. (**D**) C2C12 myoblasts treated with corylifol A (100 nM) and harvested on differentiation day 1 (D1), 2 (D2) and 3 (D3) to evaluate the expression of myogenic markers. Values are mean (*n* = 3). The images are representative of three independent experiments with similar results. Means without a common superscript differ significantly (*p* < 0.05).

Since corylifol A induced the highest MyoD transcriptional activity, we evaluated its myogenic potential in detail. C2C12 myoblasts were differentiated in the presence of the indicated concentrations (10, 50 and 100 nM) of corylifol A for three days, and then the number of differentiated myotubes were assessed by immunostaining for MHC and DAPI. The MHC-positive cells were scored with respect to the number of nuclei to quantify myoblast differentiation. Corylifol A dose-dependently increased the number of MHC-positive multinucleated myotubes, while it decreased the number of mononuclear myotubes (Figure 3A). Accordingly, corylifol A also dose-dependently enhanced the expression of myogenic factors, such as MHC, myogenin and MyoD (Figure 3B), and elevated MyoD transactivation (Figure 3C). 

To monitor the expression of myogenic factors during the differentiation period, C2C12 myoblasts were treated with 100 nM corylifol A for the three days of differentiation. As differentiation progressed, the expression of MHC and myogenin gradually increased and reached a maximum on differentiation at day three (D3), while maximal MyoD expression was observed on day two (D2) (Figure 3D). Corylifol A treatment further increased the expressions of MHC, myogenin and MyoD, as compared with their respective day control. Collectively, our results demonstrate that corylifol A enhances myogenesis in dose- (<100 nM) and time-dependent (<3 days) manners through the induction of MyoD expression as the early regulator of myoblast differentiation. 

### 2.3. Corylifol A Promotes Myogenesis via p38 MAPK Activation

**Figure 4:**
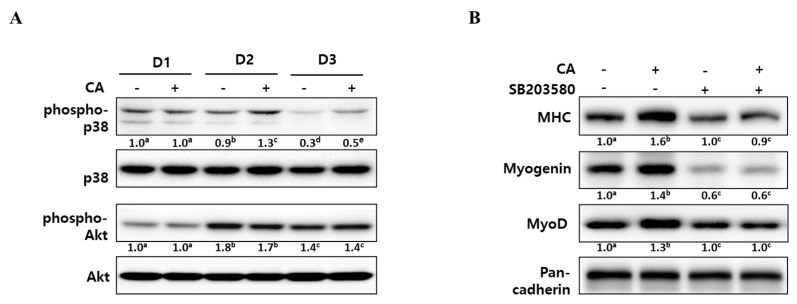
Effect of corylifol A on p38 MAPK activation during myogenesis. (**A**) C2C12 cells were treated with corylifol A (CA, 100 nM) during differentiation and subjected to Western blot analysis on differentiation day 1 (D1), 2 (D2) or 3 (D3). The levels of phospho-p38 and Akt were normalized against the respective total protein level. (**B**) C2C12 cells were pretreated with SB203580 (10 µM) prior to corylifol A and then differentiated in DM for 2 days. Cell lysates were subjected to Western blot analysis. Values are mean (*n* = 3). The images are representative of three independent experiments with similar results. Means without a common superscript differ significantly (*p* < 0.05).

To elucidate the underlying mechanisms of corylifol A for myogenesis, we checked the level of phospho-p38 mitogen-activated protein kinase (p38 MAPK) and phospho-protein kinase B (Akt), as these are known to be regulatory kinases [30,31] during differentiation. As shown in Figure 4A, the level of phosphorylated p38 MAPK was steady for two days after DM treatment and then slowly declined. The addition of corylifol A (100 nM) further sustained p38 MAPK activation, as compared with the corresponding control. In the case of the Akt pathway, phosphorylated Akt levels were maximal on D2 but were not affected by corylifol A during myoblast differentiation, compared with the respective day control.

To confirm the role of p38 MAPK in corylifol A-induced myogenesis, C2C12 myoblasts were pretreated with SB203580, a pyridinyl imidazole inhibitor of p38 MAPK, and then differentiated in the presence of corylifol A. Treatment with SB203580 showed that suppressed expression of myogenic markers could not be restored by corylifol A (Figure 4B). Immunostaining results were consistent with protein expression data for myogenic markers and showed that the number of MHC-positive multinucleated myotubes were decreased by SB203580 and were not restored by corylifol A (Supplementary Figure S1). Taken together, corylifol A induced myogenesis through the activation of the p38 MAPK pathway that is known to be required for the process.

### 2.4. Corylifol A Protects against Dexamethasone-Induced Muscle Atrophy in Vitro

To investigate the protective effect of corylifol A against muscle atrophy, fully differentiated myotubes were further incubated with dexamethasone to induce atrophy. A synthetic glucocorticoid, dexamethasone, has been well-known to induce muscle atrophy in vitro and in vivo [10,32]. In the inflammatory environment that follows dexamethasone treatment, the activation of the NF-κB pathway is essential for the expression of myostatin and muscle-specific E3 ligases, such as the muscle atrophy F-box (MAFbx/atrogin-1) and muscle RING finger 1 (MuRF1), to degrade muscle proteins [33]. 

**Figure 5:**
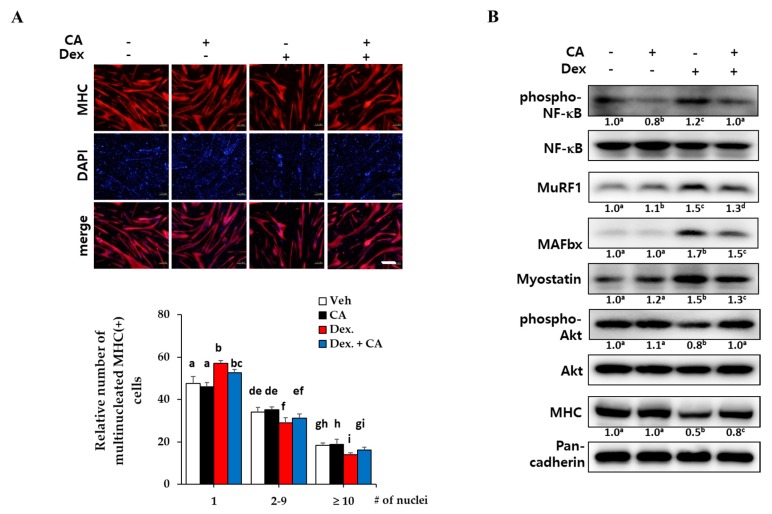
Protective effects of corylifol A against dexamethasone-induced myotube atrophy in vitro. C2C12 myoblasts were differentiated for 3 days, followed by treatment with dexamethasone (Dex, 1 µM) along with vehicle DMSO or corylifol A (CA, 100 nM) for an additional 48 h. (**A**) Collected cells were fixed and immunostained with MHC (red) and DAPI (blue) (scale bar = 200 µm). (**B**) Cell lysates were subjected to Western blot analysis to analyze phosphorylated NF-κB, E3 ligases, myostatin, phosphorylated Akt and MHC. Values are mean (*n* = 3). The images are representative of three independent experiments with similar results. Means without a common superscript differ significantly (*p* < 0.05).

As expected, dexamethasone treatment (1 µM) greatly decreased the number of MHC-expressing multinucleated myotubes, but a 48-h treatment with 100 nM corylifol A protected against myotube loss (Figure 5A). Next, we measured the levels of muscular catabolic and anabolic factors in dexamethasone-damaged myotubes. Dexamethasone activated NF-κB and increased the expression of myostatin and E3 ligases (catabolic mechanism), as compared with the vehicle control. Cotreatment with dexamethasone and corylifol A diminished their expression, as compared with the dexamethasone control (Figure 5B). 

The PI3K/Akt pathway has been shown to be activated by stimuli such as insulin and to be a central anabolic pathway leading to increased skeletal muscle mass and size [34]. As shown in Figure 5B, dexamethasone decreased the expression of MHC and the phosphorylated Akt compared to the control, but corylifol A rescued the expression of these anabolic factors. These data suggested that corylifol A protects against dexamethasone-induced muscle atrophy in vitro by contributing to the inhibition of catabolic pathways and by the activation of the anabolic pathway in muscles.

## 3. Discussion

Quiescent muscle satellite cells are activated by various stimuli and undergo myogenesis to form muscle fibers and regenerate skeletal muscle. Myogenesis is a multistage process by which different muscle-specific regulatory factors like MyoD, myogenic regulatory factor (Myf)-5, myogenin and myosin heavy chain (MHC), are expressed at different times during the myoblast differentiation [29,35,36]. MyoD and Myf-5 act as initial transcriptional regulators of myogenesis. In particular, MyoD heterodimerizes with E proteins and cooperates with myocyte enhancer factor (MEF)-2 family transcription factors to express the target gene, myogenin. Subsequently, myogenin induces relevant muscle specific factors like MHC, leading to terminal differentiation [37]. 

In this study, we purified eight compounds from an ethanol extract of *P. corylifolia* (EPC) to identify new myogenic agents. We identified corylifol A as a promising component of EPC that stimulated myogenesis in an *in vitro* experimental model using C2C12 myoblasts. Corylifol A induced the highest MyoD transcriptional activity among the eight PC compounds. It increased MHC-positive multinucleated myotubes together with increased expressions of myogenic factors, such as MHC, myogenin and MyoD (Figure 2 and Figure 3). 

At an early stage of myogenesis, p38 mitogen-activated protein kinase (p38 MAPK) mediates the phosphorylation of E proteins to facilitate heterodimerization with MyoD followed by the expression of muscle-specific genes and the prevention of premature myogenesis in activated satellite cells [38,39]. As shown in Figure 4, corylifol A enhanced the phosphorylation level of p38 MAPK on differentiation day 2 (D2) and further sustained the level on D3 to stimulate myogenesis. Another myogenic kinase, Akt was not affected by corylifol A.

To mimic muscle atrophy in vitro, differentiated myotubes were treated with dexamethasone, a synthetic glucocorticoid, for two days. Dexamethasone decreased the number of MHC-expressing multinucleated myotubes, which is a hallmark of muscle atrophy in vitro [10]. Corylifol A also protected against the impaired myotube formations, as compared with dexamethasone control (Figure 5A). As another strategy to overcome muscle atrophy, the regulation of muscle protein metabolism has been considered [6]. In a muscle atrophic environment, NF-κB-mediated E ubiquitin ligases (MuRF1 and MAFbx) are responsible for proteasomal degradation of muscle protein. Myostatin is a major negative regulator of skeletal muscle mass by acting on muscle myokine to inhibit myogenesis. As excessive myostatin accelerates muscle atrophy, inhibitors of myostatin have been attracting attention as potential targets for muscle atrophy therapeutics [6,40]. Dexamethasone increased expression levels of NF-kB-mediated E3 ligases and myostatin, indicating muscle atrophy in vitro. Corylifol A suppressed the expression of phosphorylated NF-κB, MuRF1, MAFbx and myostatin in myotubes, as compared with dexamethasone alone (Figure 5B). On the other hand, the PI3K/protein kinase B (Akt) pathway has been considered to be not only involved in the process of skeletal muscle synthesis but also required for cell survival during myogenesis. The decreased phosphorylation of Akt by dexamethasone treatment was restored by corylifol A, accompanied by increased expression of MHC. 

Several flavones exert beneficial effects on muscle maintenance. Apigenin, abundant in parsley and celery, was reported to promote myogenesis and prevent muscle weakness via Prmt7-PGC-1α-GPR56 and Prmt7-p38-myoD pathways [41]. Luteolin also prevents lipopolysaccharide-induced muscle atrophy, partly through the regulation of MAFbx expression in vitro [7]. Resveratrol and isoflavones such as genistein and daidzein are also known to have the protective effect against muscle atrophy [42,43]. Despite numerous attempts to discover agents that can inhibit proteasomal degradation and activate protein synthesis of skeletal muscle, further investigations are ongoing for their therapeutic applications.

Corylifol A, an isoflavone purified from *P. corylifolia,* has diverse physiological activities, including phytoestrogenic [44], antioxidative [18], antihepatoma [45] and hepatoprotective activities [46]. In an earlier study, we isolated a meroterpene compound, bakuchiol, a primary component of *P. corylifolia* (PC), and disclosed its myogenic potential in C2C12 myoblasts [23]. Here, we evaluated the myogenic activity of flavonoids and chalcones isolated from PC and identified corylifol A as the most effective ingredient. Corylifol A activates the differentiation of myoblasts and suppresses muscle degradation in dexamethasone-induced muscle atrophic conditions.

## 4. Materials and Methods

### 4.1. Preparation of the Ethanol Extracts of P. corylifolia L. (PC) and Purification of Flavonoids and Chalcones

PC seeds were purchased from the Jinheung Herbal Medicine Market (Seoul, Korea) and deposited as voucher specimen (No. SPH-13003) in the Herbarium of Sookmyung Women’s University. The dried and powdered seeds were extracted with ethanol at room temperature for 24 h, and the ethanol extract was partitioned with water and ethyl acetate. The eight compounds were isolated from ethyl acetate soluble fractions, as reported previously [16,17,21]. Their structures were elucidated by the analysis of infrared spectroscopy, mass and NMR spectroscopic data, as follows [13,18], bavachinin (**1**), isobavachromene (**2**), bavachalcone (**3**), corylin (**4**), bavachin (**5**), isobavachalcone (**6**), corylifol A (**7**) and neobavaisoflavone (**8**).

### 4.2. Cell Culture

C2C12 myoblasts (American Type Culture Collection, Manassas, VA, USA) were maintained in growth medium (GM) composed of Dulbecco’s Modified Eagle’s Medium (DMEM) containing 15% fetal bovine serum (Gibco BRL Life Technology, Grand island, NY, USA); 100 U/mL penicillin and 100 µg/mL streptomycin (Life Technologies, Frederick, MD, USA). When cells reached 95% confluence, differentiation of myoblasts was initiated by supplementation with differentiation medium (DM) composed of DMEM containing 2% horse serum (differentiation day 0: D0). At 3 days post-DM treatment (D3), cells were subjected to analytical experiments and image analysis. To investigate potential effects of corylifol A on muscle atrophy, differentiated myotubes were treated with corylifol A (100 nM) for 3 h prior to dexamethasone (1 µM; Sigma-Aldrich, St. Louis, MO, USA) treatment. 

### 4.3. Immunostaining of MHC

C2C12 myoblasts and differentiated myotubes were fixed with 4% paraformaldehyde for 20 min and permeabilized with 0.1% Triton X-100 for 20 min in phosphate-buffered saline (PBS). After washing with PBS, cells were stained with antibody against myosin heavy chain (MHC) (MAB4470; R&D Systems, Minneapolis, MN, USA) overnight at 4 °C, followed by an Alexa Fluor 568-conjugated secondary antibody (Life Technologies, Carlsbad, CA, USA). Nuclei were counterstained with 4′-6-diamidino-2-prenylindole (Sigma-Aldrich, St. Louis, MO, USA). Immunofluorescence was observed and captured using a fluorescence microscope (Olympus, Tokyo, Japan). Red fluorescence indicates MHC expression, and blue fluorescence indicates nuclei. The MHC-positive cells were grouped according to their number of nuclei in several fields and then presented as the relative cell counts of each group. 

### 4.4. MyoD-Reporter Gene Assay

To investigate the transcriptional activity of MyoD, C2C12 myoblasts were seeded in 24-well plates at a density of 2 × 10^4^ cells per well. Cells were transiently transfected with MyoD-responsive reporter 4RTK-luciferase (RTK-luc) and pBP-MyoD constructs [10] using lipofectamine LTX (Invitrogen, Calsbad, CA, USA) and incubated for 24 h. The transfected cells were treated with test samples for 48 h. Cell lysates were subjected to luciferase activity assay using a luciferase assay kit (Promega, Madison, WI, USA). Results are presented as relative luciferase activity divided by β-galactosidase activity.

### 4.5. Western Blot Analysis

To measure the expression of myogenic markers and E3 ligases, cells were lysed in lysis buffer (50 mM Tris, pH 7.4, 150 mM NaCl, 10% glycerol, 1.5 mM MgCl_2_, 1 mM EGTA, 1% Triton X-100, 10 mM NaF, 1mM Na_3_VO_4_, protease inhibitor cocktail (cOmplete, Roche Applied Science, Mannheim, Germany) and phosphatase inhibitor cocktail (PhosSTOP, Roche) [47]). Whole cell extracts were subjected to sodium dodecyl sulfate-polyacrylamide gel electrophoresis (SDS-PAGE), and the separated proteins were transferred onto a polyvinylfluoride (PVDF) membrane. The membrane was incubated with primary antibodies against myosin heavy chain (MHC), MyoD, myogenin, myostatin, MuRF1 (all Santa Cruz Biotechnology, Inc., Dallas, TX, USA), p38 MAPK, phospho-p38 MAPK, Akt, phospho-Akt, NF-κB, phospho-NF-κB (all Cell Signaling Technology, Inc., Beverly, MA, USA), pan-cadherin and β-actin (both Sigma-Aldrich). The protein levels were quantified using the Fusion Solo system (Vilber Lourmat, Collegien, France). 

### 4.6. Statistical Analysis

The experiments were performed at least three times. Data are expressed as mean ± standard deviation, and differences between values were assessed using one-way analysis of variance (ANOVA) followed by Duncan’s test. * *p* < 0.05 was considered statistically significant.

## 5. Conclusions

In summary, the ethanol extract of *P. corylifolia* (EPC) enhances myoblast differentiation. As the most potent component of EPC, corylifol A showed beneficial and counteracting effects on muscle atrophy via the stimulation of myogenesis and regulation of muscle protein metabolism. Corylifol A stimulates myogenesis by activating the p38 MAPK pathway and ameliorates dexamethasone-induced muscle atrophy by suppressing the NF-κB-mediated E3 ligase machinery (catabolic) and activating Akt (anabolic). This dual effect of corylifol A will provide insights into new strategies for overcoming muscle atrophy. 

## Supplementary Materials

Supplementary materials can be found at https://www.mdpi.com/1422-0067/21/5/1571/s1. Figure S1: Effect of corylifol A on p38 MAPK activation during myogenesis.
