# Supplementary material for: Corylifol A from Psoralea corylifolia L. Enhances Myogenesis and Alleviates Muscle Atrophy
Source: Int J Mol Sci. 2020 Feb 25;21(5):1571. doi: 10.3390/ijms21051571 (PMC7084366; doi:10.3390/ijms21051571)
Supplement: Supplementary file 1 [file ijms-21-01571-s001.pdf]

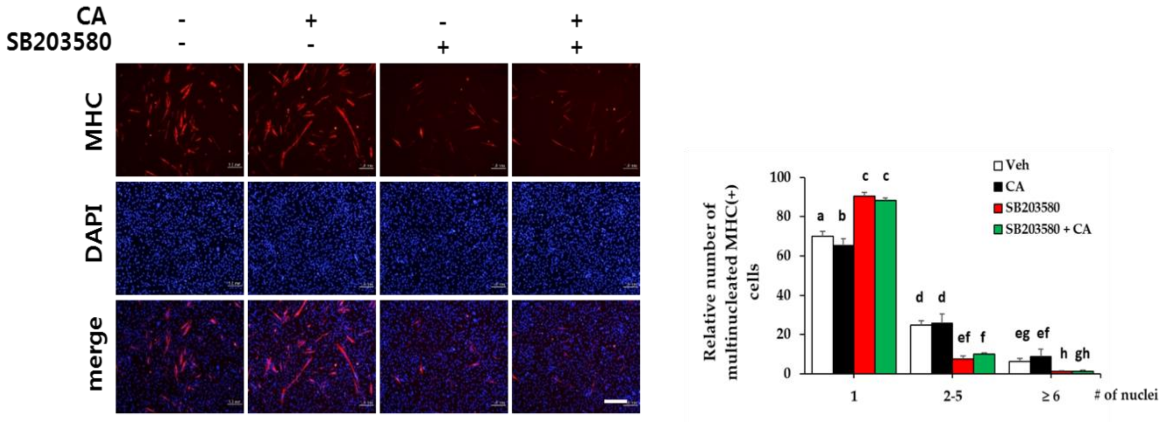

Supplementary Figure S1. Effect of corylifol A on p38 MAPK activation during myogenesis. C2C12 cells were pre-treated with SB203580 (10 μM) prior to corylifol A and then differentiated in DM for 2 days, and immunostained with MHC (red) and DAPI (blue). Scale bar = 200 μm. The images are representative of three independent experiments with similar results. Means without a common superscript differ significantly ($p < 0.05$).
